# Impact of COVID-19-related experiences on health-related quality of life in cancer survivors in the United States

**DOI:** 10.1371/journal.pone.0297077

**Published:** 2024-03-14

**Authors:** Amy K. Otto, Sarah Prinsloo, Akina Natori, Richard W. Wagner, Telma I. Gomez, Jewel M. Ochoa, Shelley S. Tworoger, Cornelia M. Ulrich, Sairah Ahmed, Jennifer L McQuade, Anita R. Peoples, Michael H. Antoni, Julienne E. Bower, Lorenzo Cohen, Frank J. Penedo

**Affiliations:** 1 Department of Public Health Sciences, Miller School of Medicine, University of Miami, Miami, FL, United States of America; 2 Sylvester Comprehensive Cancer Center, Miller School of Medicine, University of Miami, Miami, FL, United States of America; 3 Department of Palliative, Rehabilitation and Integrative Medicine, The University of Texas M.D. Anderson Cancer Center, Houston, TX, United States of America; 4 Department of Neurosurgery, The University of Texas M.D. Anderson Cancer Center, Houston, TX, United States of America; 5 Division of Medical Oncology, Department of Medicine, Miller School of Medicine, University of Miami, Miami, FL, United States of America; 6 Department of Cancer Epidemiology, H. Lee Moffitt Cancer Center and Research Institute, Tampa, FL, United States of America; 7 Huntsman Cancer Institute, University of Utah, Salt Lake City, CT, United States of America; 8 Department of Population Health Sciences, University of Utah, Salt Lake City, CT, United States of America; 9 Department of Lymphoma and Myeloma, The University of Texas M.D. Anderson Cancer Center, Houston, TX, United States of America; 10 Department of Stem Cell Transplantation and Cellular Therapy, The University of Texas M.D. Anderson Cancer Center, Houston, TX, United States of America; 11 Department of Melanoma Medical Oncology, The University of Texas M.D. Anderson Cancer Center, Houston, TX, United States of America; 12 Department of Psychology, University of Miami, Miami, FL, United States of America; 13 Department of Psychiatry and Behavioral Sciences, Miller School of Medicine, University of Miami, Miami, FL, United States of America; 14 Department of Psychology, University of California Los Angeles, Los Angeles, CA, United States of America; 15 Department of Psychiatry/Biobehavioral Sciences, University of California Los Angeles, CA, United States of America; 16 Department of Medicine, Miller School of Medicine, University of Miami, Miami, FL, United States of America; New York University, UNITED STATES

## Abstract

**Objective:**

Little evidence exists on the impact of the COVID-19 pandemic on cancer survivors, limiting recommendations to improve health-related quality of life (HRQoL) in this population. We describe survivors’ pandemic experiences and examine associations between COVID-19-related exposures, psychosocial experiences, and HRQoL.

**Methods:**

Between May 2020-April 2021, survivors completed cross-sectional questionnaires capturing COVID-19-related exposures (e.g., exposure to virus, job loss); psychosocial experiences (i.e., COVID-19-related anxiety/depression, disruptions to health care and daily activities/social interactions, satisfaction with providers’ response to COVID, financial hardship, perceived benefits of the pandemic, social support, and perceived stress management ability); and HRQoL.

**Results:**

Data were collected from *N* = 11,325 survivors in the United States. Participants were mostly female (58%), White (89%) and non-Hispanic (88%), and age 63 on average. Breast cancer was the most common diagnosis (23%). Eight percent of participants reported being exposed to COVID-19; 1% tested positive. About 6% of participants lost their jobs, while 24% lost household income. Nearly 30% avoided attending in-person oncology appointments because of the pandemic. Poorer HRQoL was associated with demographic (younger age; female; non-Hispanic White), clinical (Medicare; stage IV disease; hematologic/digestive/respiratory system cancer), and psychosocial factors (low perceived benefits and stress management ability; more disruption to health care and daily activities/social interactions; financial hardship).

**Conclusions:**

COVID-19-related stressors were associated with various psychosocial experiences in cancer survivors, and these psychosocial experiences were associated with HRQoL above and beyond demographic and clinical factors.

## Introduction

COVID-19 mitigation strategies like social distancing have significantly impacted familial, work, and social life, daily routines, and financial and emotional well-being [1–3]. Unintended consequences of mitigation strategies include disrupted, delayed, and/or avoided medical care—particularly in cancer [4]. There were estimated more than ten million cancer survivors (e.g., breast, prostate, colorectal, thyroid cancers and melanoma of the skin were major cancer types) who were in active treatment or post-treatment in the United States at the time of COVID-19 pandemic [5]. Cancer survivors are at greater risk of severe COVID-19 disease and have been disproportionately impacted by the pandemic [6–9]. They are particularly vulnerable to long-term consequences of the pandemic due to higher prevalence of preexisting health conditions and psychosocial (e.g., anxiety/depression, familial distress, financial toxicity) and physical concerns (e.g., fatigue, pain, poor sleep) associated with cancer and its treatment [10–13]. Disruptions in cancer care via delays in treatment planning, treatment initiation/continuity, and follow-up can also have detrimental consequences [14]. Survivors must cope with medical, emotional, and social concerns associated with cancer treatment or treatment response (e.g., isolation, social role strain, limited leisure activities), many of which are exacerbated by the pandemic [15, 16], further compromising HRQoL [17–20]. Conversely, potentially-protective factors like social support, having stress management skills, or perceiving benefits of a stressful situation, may buffer the impact of the pandemic on HRQoL and symptom burden [21–23].

The purpose of our research was to examine whether the impact of the pandemic on patients of all cancer types, measured by one comprehensive measure, would yield unique insights into survivors experiences. Our interest in the experience of surviving cancer while surviving a pandemic specifically included an exploration of resilience factors that we thought may contribute to a more complete picture. We hypothesized that the impact of COVID-19 would be more severe in patients who were more vulnerable to the changes in lifestyle factors required by the pandemic (social support, financial exposures, healthcare-related exposures). We also hypothesized that more adverse COVID-19 experiences would be associated with worse well-being and HRQoL, and that potential psychosocial protective factors would be associated with better HRQoL. Lastly, we explored whether our findings in a large sample of survivors would mimic those found in smaller samples.

Many studies have been published addressing the implications of the pandemic for cancer survivors, however, the prior studies have been limited by small sample size, a focus on single cancer types, and not necessarily representing large numbers of people across diverse ethnic, financial, and social backgrounds limiting the generalizability of the findings. A total of 218 peer reviewed paper were identified through a PubMed search using the MeSH terms including neoplasms, quality of life, and COVID-19. All studies conducted in the United States to assess health-related quality of life during the COVID019 pandemic among cancer survivors were eligible for full-text review. Only 12 studies remained after screening and eligibility assessment, and most studies (n = 7) focused on a single cancer type (S1 Fig). Prior studies did find lower QoL of cancer survivors who contracted COVID-19 and even who did not get COVID and were compared to a reference group [23, 24]. In addition, many studies were conducted using multiple measures, each measure determining a specific set of symptoms [24–26]. Because of the systemic nature of most QoL outcomes though, it is important to capture a more complete experience. One way to do this is by utilizing a single, comprehensive measure with multiple QoL variables rather than multiple measures to draw conclusions for a given population. In addition, no studies to date have examined resilience factors as they relate to QoL and to buffering the stress of COVID-19. By exploring the impact of the pandemic on a large sample of cancer survivors with different cancer types and demographic backgrounds, and exploring both COVID-specific stressors, resilience factors, and QoL we sought to provide a more comprehensive understanding of the effects of the pandemic on cancer survivors.

This study (1) described the initial COVID-19-related experiences and psychosocial responses of cancer survivors; (2) examined associations between those experiences and HRQoL and several domains of COVID-19-related psychosocial well-being; and (3) evaluated the association between these well-being domains and HRQoL in a large, well-characterized sample of cancer survivors.

## Participants and methods

### Participants

Participants were recruited from two NCI-designated cancer centers in the US: The University of Texas MD Anderson Cancer Center (MDACC) and University of Miami Sylvester Comprehensive Cancer Center (UM). Inclusion criteria were: ≥18 years old; English- or Spanish-speaking; clinical visit within past 5 years; and ICD-10 confirmed cancer diagnosis. Potentially eligible cancer survivors were identified through consent-to-contact lists at both institutions (n = 93,735 at MDACC and n = 8,379 at UM) (see Supplementary materials and S2 Fig).

### Procedures

Procedures conformed to US Federal Policy for the Protection of Human Subjects and were approved by the study sites’ Institutional Review Boards (MDACC; IRB #2020–0508 and UM; IRB #202000450). Initial contact about the survey was made via email at MDACC and via patient portal message at UM. Cancer survivors who agreed to participate first completed an electronic informed consent through a REDCap’s eConsent Framework. Signed informed consent was saved as a PDF in the REDCap and a copy of the certified consent was sent to a participant. Following the informed consent, participant were able to complete a questionnaire including three sections: (1) exposures and practical experiences related to the COVID-19 pandemic including testing, serostatus, loss of family/friends, and isolation (“COVID-related exposures”); (2) thoughts, experiences, and emotions regarding the COVID-19 pandemic (“COVID-related Psychosocial and Practical Experiences questionnaire,” or “COVID-PPE”); and (3) HRQoL. Questionnaires were completed between May 2020-April 2021.

### Measures

#### Demographic/clinical characteristics

The following were extracted from electronic health records: age; gender, racial/ethnic group (i.e., Hispanic of any race; non-Hispanic Black; non-Hispanic White; or non-Hispanic of another race, including American Indian/Alaska Native, Asian, Multiracial, Native Hawaiian/Pacific Islander, or self-defined “Other”); insurance status; primary cancer site/stage; and number of cancer diagnoses.

#### COVID-related exposures

Nineteen items measured viral exposure; testing and results; hospitalization due to COVID-19; COVID-19 symptoms in self, family, or friends; death of family/friends; risk factors (e.g., comorbid medical conditions, travel, work in a hospital/nursing home); social isolation; and COVID-19 effects on finances, daily life, and health care. Exposures were selected based on the recommendations of content experts from UM, MDACC, and the University of California Los Angeles—including clinical psychologists, licensed clinical social workers, and physicians working with cancer patients during the pandemic—as well as the emerging public health literature.

#### COVID-PPE

COVID-19-specific impacts were assessed using the COVID-PPE questionnaire, which consists of 37-item measure assessing subjective impact on several psychosocial domains on a scale of 0 = *Strongly Disagree* to 4 = *Strongly Agree*. This measure was developed by experts in clinical psychology, social work, and oncology from UM, MDACC, and the University of California Los Angeles, drawing from published measures assessing the impact of pandemics (e.g., H1N1) and major stressors like 9/11, instruments commonly used in cancer populations [27–29], and feedback from cancer patients, survivors, and family members. See Sáez-Clarke et al. (in press) for additional details of the development and psychometric evaluation of the COVID-PPE [30].

Internal consistency for the COVID-PPE subscales was adequate: Anxiety Symptoms (α = .85); Depression Symptoms (α = .89); Health Care Disruptions (α = .73); Satisfaction with Provider Response to the pandemic (α = .81); Disruption to Daily Activities/Social Interactions (α = .65); Financial Hardship (α = .80); Perceived Benefits of the pandemic (α = .89); Social Support (α = .61); and Perceived Stress Management Skills (α = .81). A higher subscale score indicates a higher level of the construct being assessed. Due to a technical issue, the Anxiety Symptoms and Depression Symptoms subscales and one item from the Disruption to Daily Activities/Social Interactions subscale were not displayed in the Spanish version of the questionnaire at MDACC, resulting in missing data on these items for n = 84 patients.

#### Health-related quality of life

The Functional Assessment of Cancer Therapy-7 (FACT-G7) [31] assessed overall HRQoL. FACT-G7 is a validated shorten version of the functional assessment of cancer therapy-general (FACT-G) which is a patient-reported outcome measure used to assess health-related quality of life in patients undergoing cancer therapy. This assessment consists of 7 items which are the highest priority cancer-related symptoms and concerns among 27 items in FACT-G and measures physical, emotional, and functional well-being in the past 7 days. The total scores range between 0 and 28, and with higher scores indicating better HRQoL. The internal consistency for FACT-G7 was adequate (α = .80).

### Statistical analysis

Descriptive statistics were calculated for key study variables. Effects of selected COVID-19-related exposures on COVID-PPE subscales and HRQoL were examined via independent-samples *t*-tests and linear regression, with Bonferroni correction for multiple comparisons (*p* < .0002 accepted as statistically-significant). Hierarchical linear regression modeled associations between COVID-PPE subscales and HRQoL, adjusting for demographic/clinical characteristics. Variables were entered in four steps: (1) demographics; (2) clinical characteristics; (3) COVID-PPE subscales conceptualized as psychosocial protective factors: Satisfaction with Provider Response, Perceived Benefits, Social Support, and Perceived Stress Management Ability; and (4) COVID-PPE subscales conceptualized as psychosocial risk factors: Health Care Disruption, Disruption to Daily Activities, and Financial Hardship. The Depression and Anxiety Symptoms subscales of the COVID-PPE were not included in this model due to overlap with items on the FACT-G7.

## Results

### Descriptive statistics

11,325 cancer survivors completed questionnaires (~14% recruitment rate; see supplemental materials for S2 Fig). Table 1 lists participant demographics/clinical characteristics; Table 2 lists descriptives for COVID-19-related exposures, COVID-PPE subscales, and HRQoL. Only 1% of the sample reported testing positive for COVID-19, and of those positive, 17% (0.2% of total sample) reported being hospitalized due to COVID-19. About 7% reported that a family/household member tested positive, and 33% that a friend, coworker, or neighbor tested positive. About 1% had ≥1 family/household member, and 5% had ≥1 friend, coworker, or neighbor die from COVID-19. About 6% reported losing their job due to COVID-19, 17% reported their spouse/partner lost their job, and 24% of reported decreased household income. Thirty percent and 43% reported deciding not to attend an in-person cancer care or general medical care appointment, respectively, due to COVID-19. Over half (56%) participated in telehealth; on average, participants were moderately satisfied with telehealth appointments (~3 on 0–4 scale).

**Table 1:** Sample demographic/clinical characteristics (N = 11,325).

Variable	M	SD	Variable	n	%
**Age**	63.38	12.60	Cancer Stage		
	n	%	Stages 0-III	8759	77.34
**Gender**			Stage IV	2566	22.66
** Male**	4818	42.54	Number of Cancer Diagnoses (M, SD)	1.39	0.64
** Female**	6507	57.46	1	7770	68.61
**Race**			2	2850	25.17
** White**	10030	88.57	3 or more	705	6.23
** Black**	493	4.35	Primary Cancer Site		
** Asian**	240	2.12	Breast	2557	22.58
** American Indian/Alaska Native**	39	0.34	Hematologic	1657	14.63
** Multiracial**	13	0.11	Genital system, male	1272	11.23
** Other**	424	3.74	Digestive system	1098	9.70
** Unknown/refused/missing**	74	0.65	Genital system, female	868	7.66
**Ethnicity**			Respiratory system	682	6.02
** Hispanic/Latino**	1123	9.92	Other	2841	25.09
** Non-Hispanic/Latino**	10016	88.44	Urinary tract	613	5.41
** Unknown/refused/missing**	186	1.64	Head and Neck	588	5.19
**Racial/Ethnic Group**			Endocrine	432	3.81
** Hispanic/Latino, any race**	1123	9.92	Soft tissue	388	3.43
** Non-Hispanic Black**	481	4.25	Melanoma	369	3.26
** Non-Hispanic White**	9173	81.00	Nervous system	187	1.65
** Non-Hispanic, all other races**	345	3.05	Neuroendocrine	137	1.21
** Unknown/refused/missing**	203	1.79	Bone/skeletal system	105	0.93
**Preferred Language**			Eye	38	0.34
** English**	11211	98.98	Unknown	350	3.09
** Spanish**	114	1.01	Ill-defined	238	2.10
**Health Insurance Type**			Metastatic, unknown primary	112	0.99
** Employer/union or individual plan**	5824	51.42			
** Medicare/Medicaid/other governmental**	5226	46.15			
** Uninsured**	166	1.47			
** Other/Unknown**	109	0.96			

**Table 2:** Descriptives for COVID-19 exposures, COVID-PPE subscales, and HRQoL (FACT-G7).

**COVID-19 EXPOSURES**		
**Personal Exposures**	**n**	**%**
**Exposed to someone with COVID-19**	881	7.78
**Tested for COVID-19**	3385	29.89
**Tested positive for COVID-19**	161	1.42
**Tested positive and have current symptoms**	38	0.34
**Hospitalized for COVID-19**	27	0.24
	M	SD
**Nights hospitalized**	8.88	8.37
**Family/Friend Exposures**	n	%
**Family/household member tested positive for COVID-19**	773	6.83
**Family/household member died of COVID-19**	100	0.88
**Friend/coworker/neighbor tested positive for COVID-19**	3724	32.88
**Friend/coworker/neighbor died of COVID-19**	588	5.19
	M	SD
**Number of family/household members tested positive**	1.80	1.74
**Number of friends/coworkers/neighbors tested positive**	3.76	5.97
**Risk Factors & Current Symptoms**	n	%
**COVID-19 risk factors:**		
** Age ≥65**	5965	52.67
Has comorbidities (besides cancer)^a^	4799	42.38
** Travel to COVID-19 hotspots/international travel**	612	5.43
** Visited/works in nursing home/hospital**	1334	11.78
** Current symptoms (regardless of COVID-19 test):**		
** Fever**	367	3.24
** Dry cough**	707	6.24
**Shortness of breath**	764	6.75
**Impact on Finances & Daily Life**	n	%
**Lost job/income due to COVID-19**	691	6.10
**Spouse/partner lost job/income due to COVID-19**	1961	17.32
**If employed, currently…**		
** Working from home**	2504	22.11
** Commuting**	2771	24.47
** N/A**	5863	51.77
**Household income…**		
** Decreased**	2751	24.29
Increased	134	1.18
** Not changed**	8344	73.68
**How often outside home?**		
** No time**	1222	10.79
** Once a week**	3346	29.55
** Every 2–3 days**	3647	32.20
** Normal routine**	3033	26.78
**Accomplishing…**		
** More**	1466	12.94
** Less**	6369	56.24
** The same**	3430	30.29
**Impact on Health Care**	n	%
**Decided not to attend in-person general medical appointment**	4845	42.78
**Decided not to attend in-person cancer care appointment**	3364	29.70
**Decided not to seek emergency/urgent care**	893	7.89
**Participated in telehealth**	6330	55.89
	M	SD
**Telehealth appointments for general medical care**	1.30	1.84
**Telehealth appointments for cancer care**	1.21	1.86
**Total telehealth appointments**	2.40	2.33
Satisfaction with general medical care telehealth^b^	2.98	1.12
Satisfaction with cancer care telehealth^b^	3.00	1.14
COVID-PPE Subscales^c^	M	SD
**Anxiety Symptoms**	2.33	0.92
**Depression Symptoms**	1.74	0.98
**Health Care Disruptions**	1.77	1.14
**Satisfaction with Provider Response**	3.05	0.83
**Disruption to Daily Activities/Social Interactions**	2.23	0.87
**Financial Hardship**	1.15	0.86
**Perceived Benefits**	2.93	0.75
**Social Support**	2.60	0.63
**Perceived Stress Management Ability**	2.66	0.60
**FACT-G7**	M	SD
FACT-G7 total score^d^	19.15	5.30

^a^Presence of specific comorbidities was not assessed; presence of any comorbidity was assessed with the following yes/no item: *Do you have any of the following risk factors or experienced symptoms associated with COVID-19…Comorbidities such as diabetes*, *hypertension*, *kidney disease*, *and/or respiratory illness (e*.*g*., *COPD*, *asthma)*.

^b^Scale: 0 = very dissatisfied to 4 = very satisfied.

^c^COVID-PPE subscale score range is 0–4. Higher scores indicate higher levels of the construct assessed (e.g., higher Financial Hardship score means higher levels of/worse financial hardship; higher Social Support score means higher levels of/better social support).

^d^FACT-G7 range is 0–28; higher scores indicate better HRQoL.

### Associations between COVID-related exposures and COVID-PPE and HRQoL

Table 3 summarizes associations of specific COVID-19-related exposures with COVID-PPE subscales and HRQoL (complete results in supplemental materials). *Personal and family/friend exposures* were generally related to higher anxiety and depression symptoms, health care disruption, disruption to daily activities/social interactions, financial hardship, and—interestingly—perceived benefits of the COVID-19 pandemic and social support. Personal and family/friend exposures were not associated with HRQoL.

**Table 3:** Effects of key COVID-19 exposures on COVID-PPE and FACT-G7.

	Anxiety Symptoms	Depression Symptoms	Health Care Disruptions	Satisfaction with Provider Response	Disruption to Daily Activities & Social Interactions	Financial Hardship	Perceived Benefits	Social Support	Perceived Stress Management Ability	FACT-G7
**Exposed to someone with COVID-19 (Y/N)**		↑				↑				
**Tested positive for COVID-19 (Y/N)**			↑							
**Family/household member tested positive (Y/N)**					↑		↑			
**Family/household member died of COVID-19 (Y/N)**	↑				↑					
**Friend/coworker/neighbor tested positive (Y/N)**	↑	↑	↑		↑	↑	↑	↑		
**Friend/coworker/neighbor died of COVID-19 (Y/N)**	↑	↑	↑		↑	↑	↑	↑		
**COVID-19 risk factors:**										
** ≥65 years old (Y/N)**	↓	↓			↓	↓		↓	↓	↑
Has comorbidities other than cancer^a^ (Y/N)	↑	↑	↑		↑	↑				↓
** Travel to COVID-19 hotspots/international travel (Y/N)**			↑		↑					
** Visited or works in nursing home/hospital (Y/N)**					↑					↓
**Lost job/income due to COVID-19 (Y/N)**		↑	↑		↑	↑				↓
**Spouse/partner lost job/income due to COVID-19 (Y/N)**	↓	↓			↓		↓	↓	↓	↑
**Decided not to attend an in-person general medical appointment (Y/N)**			↑							
**Decided not to attend an in-person cancer care appointment (Y/N)**			↑							
**Decided not to seek emergency/urgent care (Y/N)**			↑							↓
**Working from home (H) vs. commuting to work (C)**	C>H	C>H			C>H			C>H	C>H	
**Household income decreased (D) vs. other (O; i.e., same or increased)**	D>O	D>O	D>O		D>O	D>O				O>D
**Number of telehealth appointments for general medical care**	↑	↑	↑		↑	↑				↓
**Number of telehealth appointments for cancer care**				↑				↑		↓
**Total number of telehealth appointments**	↑	↑			↑	↑		↑		↓
**Satisfaction with general medical care telehealth**		↓	↓	↑	↓	↓	↑	↑	↑	↑
**Satisfaction with cancer care telehealth**		↓	↓	↑	↓	↓	↑	↑	↑	↑

*Note*. Blank cells indicate non-significant effects; effects displayed are all significant after Bonferroni correction for multiple comparisons (*p* < .0002). Up arrow (↑) indicates a positive association—i.e., either (1) those who responded “Yes” had higher scores than those who responded “No,” or (2) there was a positive relationship between the item and the outcome. Down arrow (↓) indicates a negative relationship—i.e., either (1) those who responded “No” had higher scores than those who responded “Yes,” or (2) there was a negative relationship between the item and the outcome.

#### Risk factors for COVID-19

Participants age ≥65 (vs. <65) reported lower anxiety and depression symptoms, disruption to daily activities/social interactions, and financial hardship, along with increased HRQoL. However, they also reported lower social support and perceived stress management ability. Having comorbid medical conditions besides cancer was associated with higher anxiety and depression symptoms, health care disruption, disruption to daily activities/social interactions, and financial hardship, as well as decreased HRQoL. Visiting or working in a nursing home/hospital was associated with increased disruption to daily activities/social interactions and lower HRQoL.

#### Employment/financial exposures

Participants who lost their job/source of income, who commuted to work (vs. working from home), and whose household income decreased reported greater anxiety and depression symptoms, health care disruption, disruption to daily activities/social interactions, financial hardship, and lower HRQoL. Those who commuted to work reported higher levels of social support and perceived stress management ability vs. those who worked from home. Interestingly, participants whose spouse/partner lost their job/income reported less anxiety and depression symptoms, lower disruption to daily activities/social interactions, and better HRQoL than participants whose partner did not lose their job/income; however, these participants also reported lower perceived benefits of the pandemic, lower social support, and lower perceived stress management ability.

#### Health care-related exposures

Unsurprisingly, participants who reported not attending in-person medical appointments and those who avoided emergency/urgent care reported greater disruptions to health care during the pandemic. Those who avoided emergency/urgent care also reported lower HRQoL than those who did not avoid this care. Participating in more telehealth appointments was associated with greater anxiety and depression symptoms, disruption to health care, disruption to daily activities/social interactions, and financial hardship, as well as decreased HRQoL. However, more telehealth appointments were also associated with increased satisfaction with providers’ response to COVID-19 and increased social support. Greater satisfaction with telehealth was associated with less depression symptoms, health care disruption, disruption to daily activities/social interactions, and financial hardship, along with increased satisfaction with providers’ response, perceived benefits, social support, perceived stress management ability, and HRQoL.

### Association between COVID-related psychosocial and practical experiences and HRQoL

Hierarchical regression evaluated associations between COVID-PPE subscale scores and HRQoL, adjusting for key demographic/clinical characteristics. Predictors were entered in four steps (see Table 4). Demographics alone (step 1) accounted for 2% of the variance in HRQoL. The inclusion of each additional block of variables independently accounted for significant increases in the variance of HRQoL, with step 4 (risk factors) producing the largest increase (20%). This final model accounted for a total of 31% of variance in HRQoL. In this model, poorer HRQoL was associated with younger age; female gender; identifying as non-Hispanic White (vs. non-Hispanic “Other”); having a governmental insurance plan (vs. employer/union or individual plan); having stage IV disease; having hematologic, digestive system, or respiratory system cancers (vs. breast cancer); lower perceived benefits of the pandemic and stress management ability; and higher health care disruption, disruption to daily activities/social interactions, and financial hardship.

**Table 4:** Results of hierarchical multiple regression for FACT-G7.

Predictor variable	Model 1	Model 2	Model 3	Model 4
	B	B	B	B
**Step 1**				
Age	0.047***	0.047***	0.043***	0.020***
Female gender	-0.878***	-1.026***	-1.381***	-0.881***
Racial/Ethnic Group				
Non-Hispanic White (ref.)	--	--	--	--
Non-Hispanic Black	0.342	0.273	-0.207	-0.268
Non-Hispanic Other	0.571	0.607	0.434	0.690*
Hispanic, any race	-0.107	-0.181	-0.376*	-0.078
Spanish-speaking	0.203	0.161	-0.201	0.600
Insurance				
Employer/union/individual (ref.)	--	--	--	--
Governmental	-0.891***	-0.811***	-0.710***	-0.631***
Other	-1.458***	-1.414***	-1.216***	-0.525
**Step 2**				
Stage IV disease		-0.830***	-0.849***	-0.920***
Number of cancer diagnoses		-0.214	-0.235*	-0.182
Cancer site				
Male genital (ref.)		--	--	--
Breast cancer		0.224	0.088	0.087
Hematologic		-0.917***	-0.917***	-0.788***
Female genital		-0.354	-0.364	-0.317
Digestive system		-0.966***	-0.966***	-0.937***
Respiratory system		-1.359***	-1.355***	-1.194***
Other		0.036	-0.008	0.054
Unknown		0.251	0.207	0.091
**Step 3**				
Satisfaction with Provider Response			0.090	-0.072
Perceived Benefits			-0.063	0.155*
Social Support			-0.438***	-0.119
Perceived Stress Management Ability			2.525***	1.823***
**Step 4**				
Health Care Disruption				-0.569***
Disruption to Daily Activities				-1.715***
Financial Hardship				-1.253***
F	22.382***	20.424***	55.862***	187.291***
R^2^	0.017	0.033	0.104	0.309
ΔR^2^	0.017***	0.016***	0.071***	0.204***

*p < .05

**p < .01

***p < .001

## Discussion

The COVID-19 pandemic has disproportionately affected cancer survivors. Health care systems have delayed non-urgent procedures, including post-treatment cancer screenings [13, 32], with about half of survivors experiencing a COVID-19-related health care delay [33]. Many survivors have been transitioned to telehealth; while this may facilitate care in some circumstances (e.g., improved coordination) [27], <5% of survivors used telemedicine prior to the pandemic [34]. This study evaluated how survivors’ initial experiences of the COVID-19 pandemic were associated with various domains of psychosocial well-being and HRQoL.

In our sample, 1% of survivors reported testing positive for COVID-19, with 7% indicating that a family/household member tested positive and 33% that a friend/coworker/neighbor tested positive. By the end of data collection in April 2021, US cases surpassed 32 million (~10% of the total population) [35]; that survivors reported a lower positivity rate than the general population may not be too surprising; as noted earlier, cancer survivors are at greater risk of more severe COVID-19 and may therefore have been more cautious interacting with others, reducing chances of exposure. Despite low infection rates, however, we identified significant consequences of the pandemic across several domains. Six percent of survivors lost their job, 17% had a spouse/partner lose their job, and 24% reported decreased household income. Survivors who may rely on employer-provided insurance, and who may have had to stop work or decrease time spent at work because of disease sequelae, are likely to be disproportionately affected by job/income loss. Having a job and a routine, being able to contribute financially to a household, and productivity from employment may contribute to a survivor’s sense of autonomy, purpose, and well-being.

Results showed that many survivors avoided receiving in-person medical care due to COVID-19, with 30% canceling a cancer care appointment, 43% canceling a general medical appointment, and 8% avoiding emergency/urgent care. This is consistent with other work showing 30% of breast cancer patients canceled an oncology/hematology appointment because of COVID-19, primarily due to fear about contracting the virus [36]. It remains unclear how concerning these numbers are, as an even larger proportion of participants reported participating in ≥1 telehealth appointment (56%), although it is unlikely that *all* canceled in-person appointments were replaced with telehealth (e.g., cancer screenings, procedures). When telehealth is feasible, however, survivors find it acceptable: In our sample, survivors averaged ~3 (on a 0–4 scale) for satisfaction with telehealth. In other studies, oncology patients also describe telehealth as equal to or better than in-person visits, and studies across age ranges and health specializations have reported similar results [37–39].

Associations between COVID-related exposures and psychosocial outcomes (COVID-PPE scores and HRQoL) were generally in expected directions, with a notable exception: While participants whose spouse/partner lost their job reported lower perceived benefits of the pandemic, lower social support, and lower perceived stress management ability, they also reported *less* anxiety and depression, *lower* disruption to daily activities and social interactions, and *better* HRQoL than participants whose partner did not lose their job. A partner’s job loss may mean that the survivor was able to spend more time with their partner, which may have helped to alleviate stress and reduce feelings of general disruption to social interactions—and it has been shown that having family members close in proximity yields lower levels of stress, anxiety, and depression [40]. However, the fact that a partner’s job loss was also associated with *lower* levels of social support suggests other factors may be at play; for example, it is possible that a newly-unemployed partner might take on more of the “cognitive labor” in the relationship/household—e.g., anticipating needs, decision-making, monitoring progress on tasks [41]. Cognitive labor is often invisible [41], so survivors may have benefited from this support without conscious awareness that they were receiving it.

Results also indicated that psychosocial protective factors contributed more than demographics and clinical characteristics in terms of amount of explained variance in HRQoL, but psychosocial risk factors contributed the most. The final model showed that worse HRQoL was associated with perceiving fewer benefits of the pandemic, less perceived stress management ability, more health care disruption, more disruption to daily activities/social interactions, and more financial hardship. Younger age and female gender were also associated with worse HRQoL. Responsibilities society often delegates to women, especially younger women (e.g., child care) may have been particularly difficult during the pandemic, as at-home schooling and multi-generational households became more normalized. Overall, restrictions and lifestyle changes imposed by the pandemic have diminished social network density and diversity, cohesion across social structures (e.g., neighborhood, family, friends), and overall interpersonal and institutional trust, which can all affect health outcomes [42].

### Limitations and future directions

Our cross-sectional design precludes causal inferences; rather, it provides a descriptive view of how the pandemic impacted a large, well-characterized sample of cancer survivors. Although our sample was large, it was somewhat homogeneous with regard to race/ethnicity, and the overall response rate was low (~14%), raising concerns about generalizability; however, the rate was comparable to other large-scale studies involving electronically-distributed self-report questionnaires [43–45]. As family presence can yield lower levels of stress, anxiety, and depression [46], future studies should evaluate factors such as family functioning, shared relational beliefs, and relationship quality. It should also be noted that the pandemic is still ongoing/evolving—findings from earlier on might not generalize to the present/future, especially given vaccine availability and emergence of new variants. Future work should evaluate long-term impacts of COVID-19 across multiple psychosocial risk and protective domains. Future research also should focus on building individual resilience in the areas of stress management skills and finding benefit/meaning from life’s challenges.

## Conclusions and implications

Prior to the pandemic, cancer survivors were disproportionately affected by psychosocial challenges. With the additional stressor of a pandemic and associated fear of financial insecurity, limited health care availability, and decreased social contact, it follows that these issues will be major risk factors in mental and physical health outcomes. Although our study found these risk factors were associated with lower HRQoL, it also demonstrated the protective influence of factors like perceived stress management ability—with these factors explaining a larger amount of variance in HRQoL compared to demographic and clinical factors. This suggests that practitioners should acknowledge patients’ strengths and positive experiences when evaluating risk for poor psychosocial outcomes due to COVID-19, and make referrals to the appropriate resources for those with limited protective factors. Researchers should continue collecting data as the pandemic continues (and/or dissipates); these data can continue to inform health care policy, as well as interventions and programs for cancer survivors.

## Supporting information

S1 Fig PRISMA diagram. Medline (Host: PubMed) was searched for studies published from January 2020 to November 2023 using the MeSH term (1) neoplasms, (2) quality of life, and (3) COVID-19. Eligible studies comprised those evaluating health-related quality of life among cancer patients during COVID-19 pandemic and conducted in the United States. We excluded reviews, systematic reviews/meta-analysis, or studies with sample size less than 100.
(TIF)

S2 Fig CONSORT diagram. (TIF)

S1 Table Effects of key COVID-19 exposures on psychosocial & practical experiences and FACT-G7 score. Cells highlighted in green indicate significant effects (*p* < .0002 after Bonferroni correction); non-highlighted cells indicate non-significant effects (*p* ≥ .0002).
(DOCX)

S1 Data (CSV)
