# Supplementary figures and images for: Impact of COVID-19-related experiences on health-related quality of life in cancer survivors in the United States
Source: PLoS One. 2024 Mar 14;19(3):e0297077. doi: 10.1371/journal.pone.0297077 (PMC10939216; doi:10.1371/journal.pone.0297077)

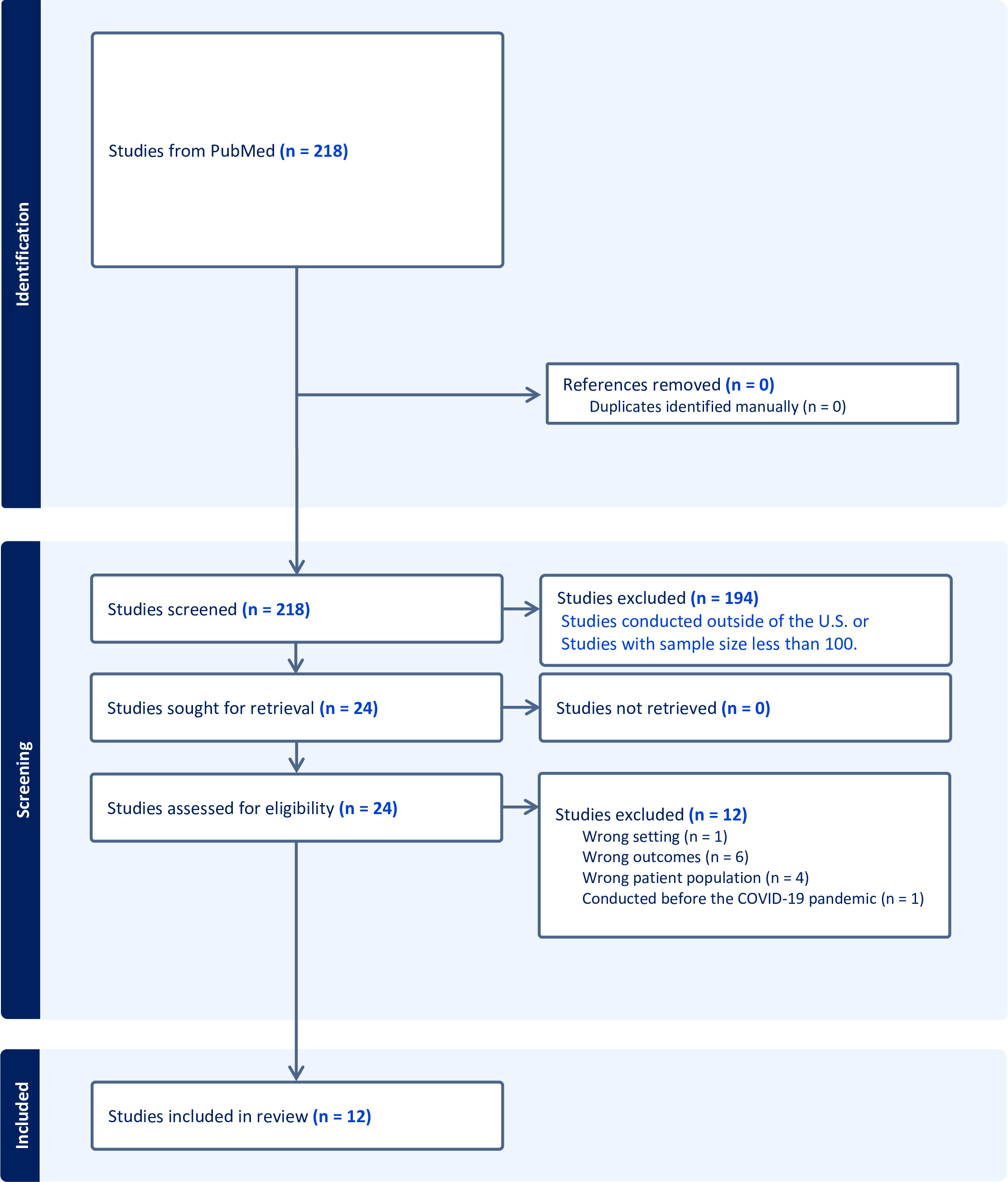

Supplement: S1 Fig — Medline (Host: PubMed) was searched for studies published from January 2020 to November 2023 using the MeSH term (1) neoplasms, (2) quality of life, and (3) COVID-19. Eligible studies comprised those evaluating health-related quality of life among cancer patients during COVID-19 pandemic and conducted in the United States. We excluded reviews, systematic reviews/meta-analysis, or studies with sample size less than 100. (TIF) [file pone.0297077.s001.tif]

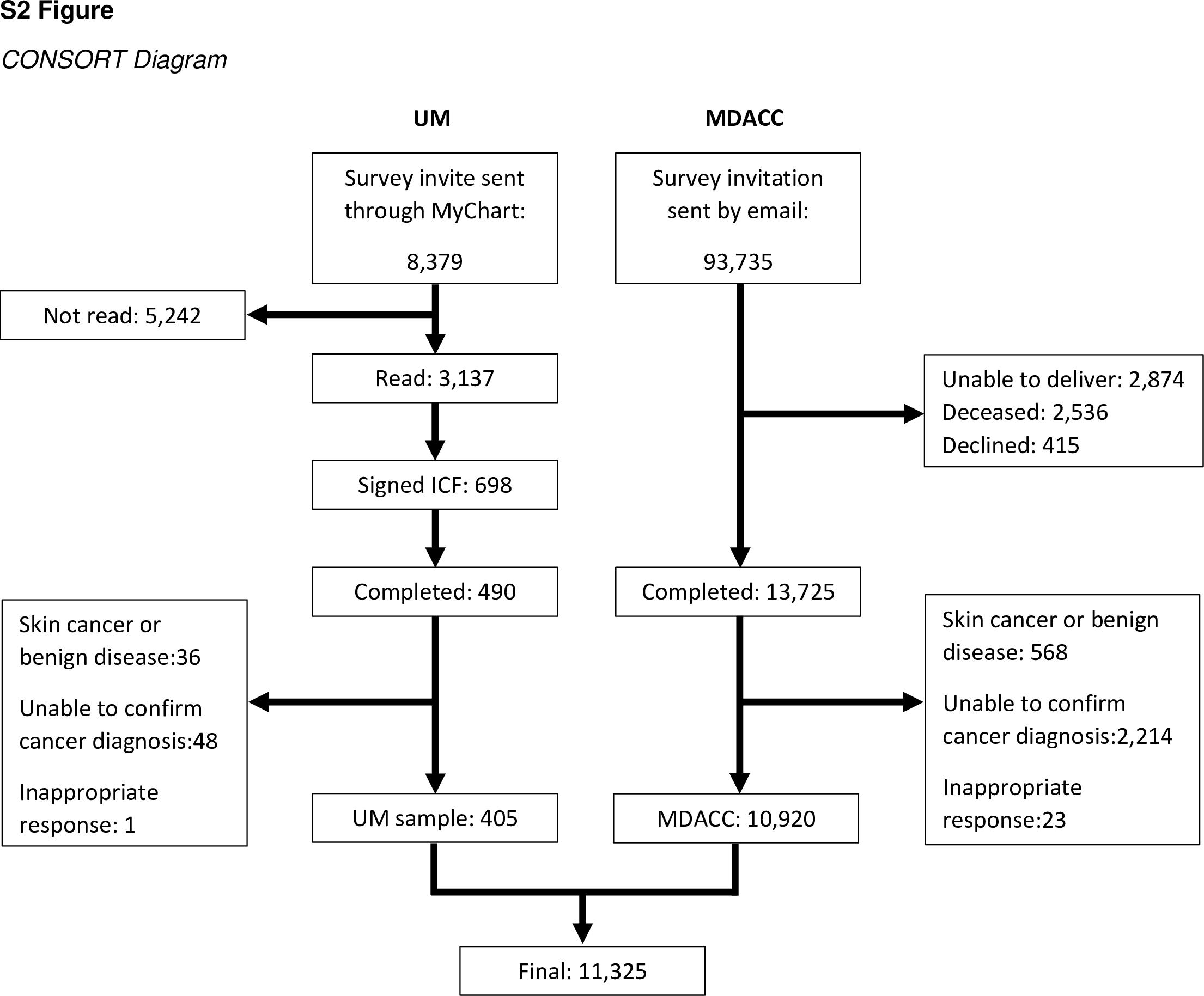

Supplement: S2 Fig — (TIF) [file pone.0297077.s002.tif]
