# Supplementary material for: Impact of COVID-19-related experiences on health-related quality of life in cancer survivors in the United States
Source: PLoS One. 2024 Mar 14;19(3):e0297077. doi: 10.1371/journal.pone.0297077 (PMC10939216; doi:10.1371/journal.pone.0297077)
Supplement: S1 Table — Cells highlighted in green indicate significant effects (p < .0002 after Bonferroni correction); non-highlighted cells indicate non-significant effects (p ≥ .0002). (DOCX) [file pone.0297077.s003.docx]

**S1 Table**

*Detailed Results for Table 3 in Main Text: Effects of Key COVID-19 Exposures on Psychosocial & Practical Experiences and FACT-G7 Score*

|  | Anxiety Symptoms | Depression Symptoms | Health Care Disruptions | Satisfaction with Provider Response | Disruption to Daily Activities & Social Interactions | Financial Hardship | Perceived Benefits | Social Support | Perceived Stress Management Ability | FACT-G7 |
| --- | --- | --- | --- | --- | --- | --- | --- | --- | --- | --- |
| Exposed to someone with COVID-19 (Y/N) | *M*_Y_=2.39, *SD*_Y_=0.90  *M*_N_=2.30, *SD*_N_=0.92  *t*(9919)=-2.80  *p*=.00517 | *M*_Y_=1.85, *SD*_Y_=1.03  *M*_N_=1.70, *SD*_N_=0.97  *t*(9912)=-4.17  *p*=.00003 | *M*_Y_=1.84, *SD*_Y_=1.14  *M*_N_=1.74, *SD*_N_=1.14  *t*(9886)=-2.31  *p*=.02096 | *M*_Y_=2.96, *SD*_Y_=0.88  *M*_N_=3.06, *SD*_N_=0.83  *t*(9869)=3.45  *p*=.00056 | *M*_Y_=2.32, *SD*_Y_=0.89  *M*_N_=2.21, *SD*_N_=0.86  *t*(9745)=-3.50  *p*=.00047 | *M*_Y_=1.31, *SD*_Y_=0.95  *M*_N_=1.11, *SD*_N_=0.84  *t*(9703)=-6.53  *p*<.00001 | *M*_Y_=2.93, *SD*_Y_=0.77  *M*_N_=2.93, *SD*_N_=0.74  *t*(9653)=-0.17  *p*=.86322 | *M*_Y_=2.64, *SD*_Y_=0.65  *M*_N_=2.60, *SD*_N_=0.63  *t*(9595)=-1.88  *p*=.06015 | *M*_Y_=2.69, *SD*_Y_=0.65  *M*_N_=2.67, *SD*_N_=0.60  *t*(9436)=-1.07  *p*=.28560 | *M*_Y_=18.86, *SD*_Y_=5.69  *M*_N_=19.30, *SD*_N_=5.22  *t*(9340)=2.20  *p*=.02760 |
| Tested positive for COVID-19 (Y/N) | *M*_Y_=2.50, *SD*_Y_=0.93  *M*_N_=2.38, *SD*_N_=0.91  *t*(3104)=-1.57  *p*=.11691 | *M*_Y_=1.99, *SD*_Y_=1.06  *M*_N_=1.79, *SD*_N_=0.98  *t*(3102)=-2.54  *p*=.01102 | *M*_Y_=2.19, *SD*_Y_=1.15  *M*_N_=1.81, *SD*_N_=1.16  *t*(3089)=-3.86  *p*=.00012 | *M*_Y_=2.93, *SD*_Y_=0.87  *M*_N_=3.14, *SD*_N_=0.82  *t*(3084)=3.01  *p*=.00265 | *M*_Y_=2.54, *SD*_Y_=0.86  *M*_N_=2.32, *SD*_N_=0.86  *t*(3047)=-3.03  *p*=.00249 | *M*_Y_=1.36, *SD*_Y_=0.98  *M*_N_=1.19, *SD*_N_=0.88  *t*(3022)=-2.29  *p*=.02221 | *M*_Y_=3.06, *SD*_Y_=0.78  *M*_N_=2.95, *SD*_N_=0.73  *t*(3011)=-1.64  *p*=.10110 | *M*_Y_=2.73, *SD*_Y_=0.73  *M*_N_=2.63, *SD*_N_=0.64  *t*(2990)=-1.81  *p*=.07027 | *M*_Y_=2.69, *SD*_Y_=0.77  *M*_N_=2.67, *SD*_N_=0.59  *t*(2939)=-0.35  *p*=.72379 | *M*_Y_=18.53, *SD*_Y_=6.30  *M*_N_=18.21, *SD*_N_=5.47  *t*(2893)=-0.66  *p*=.51198 |
| Family/household member tested positive (Y/N) | *M*_Y_=2.42, *SD*_Y_=0.92  *M*_N_=2.32, *SD*_N_=0.92  *t*(10549)=-2.68  *p*=.00744 | *M*_Y_=1.79, *SD*_Y_=1.02  *M*_N_=1.73, *SD*_N_=0.98  *t*(10541)=-1.58  *p*=.11493 | *M*_Y_=1.91, *SD*_Y_=1.18  *M*_N_=1.76, *SD*_N_=1.14  *t*(10510)=-3.57  *p*=.00036 | *M*_Y_=3.01, *SD*_Y_=0.85  *M*_N_=3.05, *SD*_N_=0.82  *t*(10492)=1.18  *p*=.23767 | *M*_Y_=2.36, *SD*_Y_=0.86  *M*_N_=2.21, *SD*_N_=0.86  *t*(10362)=-4.22  *p*=.00002 | *M*_Y_=1.23, *SD*_Y_=0.90  *M*_N_=1.14, *SD*_N_=0.85  *t*(10313)=-2.67  *p*=.00751 | *M*_Y_=3.04, *SD*_Y_=0.76  *M*_N_=2.92, *SD*_N_=0.75  *t*(10267)=-4.12  *p*=.00004 | *M*_Y_=2.66, *SD*_Y_=0.66  *M*_N_=2.60, *SD*_N_=0.63  *t*(10203)=-2.69  *p*=.00719 | *M*_Y_=2.71, *SD*_Y_=0.60  *M*_N_=2.66, *SD*_N_=0.60  *t*(10034)=-2.25  *p*=.02476 | *M*_Y_=18.88, *SD*_Y_=5.33  *M*_N_=19.21, *SD*_N_=5.28  *t*(9927)=1.55  *p*=.12080 |
| Family/household member died of COVID-19 (Y/N) | *M*_Y_=2.69, *SD*_Y_=0.73  *M*_N_=2.33, *SD*_N_=0.92  *t*(10801)=-3.79  *p*=.00015 | *M*_Y_=2.10, *SD*_Y_=0.98  *M*_N_=1.74, *SD*_N_=0.98  *t*(10793)=-3.62  *p*=.00030 | *M*_Y_=2.06, *SD*_Y_=1.03  *M*_N_=1.77, *SD*_N_=1.14  *t*(10761)=-2.46  *p*=.01401 | *M*_Y_=2.96, *SD*_Y_=0.89  *M*_N_=3.05, *SD*_N_=0.83  *t*(10743)=0.99  *p*=.32352 | *M*_Y_=2.58, *SD*_Y_=0.82  *M*_N_=2.23, *SD*_N_=0.87  *t*(10613)=-3.94  *p*=.00008 | *M*_Y_=1.33, *SD*_Y_=0.85  *M*_N_=1.15, *SD*_N_=0.86  *t*(10558)=-2.00  *p*=.04567 | *M*_Y_=3.07, *SD*_Y_=0.81  *M*_N_=2.93, *SD*_N_=0.75  *t*(10512)=-1.84  *p*=.06601 | *M*_Y_=2.71, *SD*_Y_=0.75  *M*_N_=2.60, *SD*_N_=0.63  *t*(10446)=-1.69  *p*=.09097 | *M*_Y_=2.73, *SD*_Y_=0.70  *M*_N_=2.66, *SD*_N_=0.60  *t*(10273)=-0.94  *p*=.34963 | *M*_Y_=18.22, *SD*_Y_=4.88  *M*_N_=19.17, *SD*_N_=5.30  *t*(10163)=1.63  *p*=.10274 |
| Friend/coworker/neighbor tested positive (Y/N) | *M*_Y_=2.47, *SD*_Y_=0.88  *M*_N_=2.26, *SD*_N_=0.93  *t*(10790)=-11.42  *p*<.00001 | *M*_Y_=1.86, *SD*_Y_=1.00  *M*_N_=1.68, *SD*_N_=0.97  *t*(10787)=-9.26  *p*<.00001 | *M*_Y_=,1.84 *SD*_Y_=1.14  *M*_N_=1.73, *SD*_N_=1.14  *t*(10752)=-4.78  *p*<.00001 | *M*_Y_=3.05, *SD*_Y_=0.81  *M*_N_=3.04, *SD*_N_=0.83  *t*(10734)=-0.65  *p*=.51403 | *M*_Y_=2.34, *SD*_Y_=0.84  *M*_N_=2.17, *SD*_N_=0.87  *t*(10602)=-9.67  *p*<.00001 | *M*_Y_=1.21, *SD*_Y_=0.88  *M*_N_=1.12, *SD*_N_=0.85  *t*(10547)=-5.04  *p*<.00001 | *M*_Y_=2.99, *SD*_Y_=0.73  *M*_N_=2.90, *SD*_N_=0.76  *t*(10500)= -5.87  *p*<.00001 | *M*_Y_=2.66, *SD*_Y_=0.62  *M*_N_=2.57, *SD*_N_=0.64  *t*(10435)=-6.47  *p*<.00001 | *M*_Y_=2.69, *SD*_Y_=0.60  *M*_N_=2.65, *SD*_N_=0.60  *t*(10261)=-3.16  *p*=.00157 | *M*_Y_=18.98, *SD*_Y_=5.28  *M*_N_=19.25, *SD*_N_=5.30  *t*(10151)=2.44  *p*=.01459 |
| Friend/coworker/neighbor died of COVID-19 (Y/N) | *M*_Y_=2.61, *SD*_Y_=0.88  *M*_N_=2.30, *SD*_N_=0.92  *t*(10167)=-7.74  *p*<.00001 | *M*_Y_=2.01, *SD*_Y_=0.95  *M*_N_=1.71, *SD*_N_=0.98  *t*(10158)=-6.97  *p<*.00001 | *M*_Y_=2.01, *SD*_Y_=1.18  *M*_N_=1.75, *SD*_N_=1.14  *t*(10127)=-5.29  *p*<.00001 | *M*_Y_=3.07, *SD*_Y_=0.87  *M*_N_=3.05, *SD*_N_=0.82  *t*(10110)=-0.78  *p*=.43342 | *M*_Y_=2.48, *SD*_Y_=0.81  *M*_N_=2.21, *SD*_N_=0.87  *t*(9987)=-7.09  *p*<.00001 | *M*_Y_=1.30, *SD*_Y_=0.94  *M*_N_=1.13, *SD*_N_=0.85  *t*(9934)=-4.47  *p*=.00001 | *M*_Y_=3.10, *SD*_Y_=0.73  *M*_N_=2.92, *SD*_N_=0.75  *t*(9891)=-5.67  *p*<.00001 | *M*_Y_=2.71, *SD*_Y_=0.63  *M*_N_=2.60, *SD*_N_=0.63  *t*(9831)=-4.24  *p*=.00002 | *M*_Y_=2.72, *SD*_Y_=0.61  *M*_N_=2.66, *SD*_N_=0.60  *t*(9668)=-1.97  *p*=.04853 | *M*_Y_=18.48, *SD*_Y_=5.10  *M*_N_=19.27, *SD*_N_=5.29  *t*(9569)=3.37  *p*=.00076 |
| COVID-19 risk factors: |  |  |  |  |  |  |  |  |  |  |
| ≥65 years old (Y/N) | *M*_Y_=2.26, *SD*_Y_=0.91  *M*_N_=2.41, *SD*_N_=0.92  *t*(10860)=8.90  *p*<.00001 | *M*_Y_=1.65, *SD*_Y_=0.95  *M*_N_=1.84, *SD*_N_=1.01  *t*(10851)=10.34  *p*<.00001 | *M*_Y_=1.75, *SD*_Y_=1.12  *M*_N_=1.79, *SD*_N_=1.16  *t*(10820)=1.92  *p*=.05476 | *M*_Y_=3.03, *SD*_Y_=0.84  *M*_N_=3.07, *SD*_N_=0.81  *t*(10801)=2.60  *p*=.00925 | *M*_Y_=2.18, *SD*_Y_=0.86  *M*_N_=2.29, *SD*_N_=0.88  *t*(10671)=6.86  *p*<.00001 | *M*_Y_=1.01, *SD*_Y_=0.79  *M*_N_=1.31, *SD*_N_=0.90  *t*(10615)=17.94  *p*<.00001 | *M*_Y_=2.91, *SD*_Y_=0.73  *M*_N_=2.95, *SD*_N_=0.77  *t*(10566)=2.44  *p*=.01483 | *M*_Y_=2.56, *SD*_Y_=0.63  *M*_N_=2.65, *SD*_N_=0.63  *t*(10501)=7.06  *p*<.00001 | *M*_Y_=2.64, *SD*_Y_=0.59  *M*_N_=2.69, *SD*_N_=0.62  *t*(10327)=3.77  *p*=.00017 | *M*_Y_=19.49, *SD*_Y_=5.09  *M*_N_=18.75, *SD*_N_=5.51  *t*(10216)=-7.07  *p*<.00001 |
| Has comorbidities other than cancer^a^ (Y/N) | *M*_Y_=2.45, *SD*_Y_=0.89  *M*_N_=2.24, *SD*_N_=0.94  *t*(10805)=-11.61  *p*<.00001 | *M*_Y_=1.85, *SD*_Y_=0.97  *M*_N_=1.66, *SD*_N_=0.98  *t*(10796)=-9.76  *p*<.00001 | *M*_Y_=1.86, *SD*_Y_=1.14  *M*_N_=1.70, *SD*_N_=1.13  *t*(10767)=-7.39  *p*<.00001 | *M*_Y_=3.05, *SD*_Y_=0.82  *M*_N_=3.04, *SD*_N_=0.83  *t*(10749)=-0.31  *p*=.75673 | *M*_Y_=2.31, *SD*_Y_=0.85  *M*_N_=2.17, *SD*_N_=0.87  *t*(10618)=-8.23  *p*<.00001 | *M*_Y_=1.20, *SD*_Y_=0.87  *M*_N_=1.10, *SD*_N_=0.85  *t*(10562)=-5.66  *p*<.00001 | *M*_Y_=2.92, *SD*_Y_=0.74  *M*_N_=2.93, *SD*_N_=0.76  *t*(10514)=0.63  *p*=.53058 | *M*_Y_=2.59, *SD*_Y_=0.63  *M*_N_=2.60, *SD*_N_=0.64  *t*(10449)=1.07  *p*=.28485 | *M*_Y_=2.65, *SD*_Y_=0.60  *M*_N_=2.67, *SD*_N_=0.61  *t*(10275)=1.78  *p*=.07581 | *M*_Y_=18.35, *SD*_Y_=5.40  *M*_N_=19.77, *SD*_N_=5.14  *t*(10164)=13.49  *p*<.00001 |
| Travel to COVID-19 hotspots/international travel (Y/N) | *M*_Y_=2.43, *SD*_Y_=0.89  *M*_N_=2.32, *SD*_N_=0.92  *t*(10824)=-2.75  *p*=.00594 | *M*_Y_=1.88, *SD*_Y_=0.97  *M*_N_=1.73, *SD*_N_=0.98  *t*(10815)=-3.52  *p*=.00044 | *M*_Y_=1.96, *SD*_Y_=1.15  *M*_N_=1.76, *SD*_N_=1.14  *t*(10785)=-4.09  *p*=.00004 | *M*_Y_=3.00, *SD*_Y_=0.85  *M*_N_=3.05, *SD*_N_=0.83  *t*(10767)=1.52  *p*=.12893 | *M*_Y_=2.39, *SD*_Y_=0.84  *M*_N_=2.22, *SD*_N_=0.87  *t*(10636)=-4.60  *p*<.00001 | *M*_Y_=1.25, *SD*_Y_=0.87  *M*_N_=1.14, *SD*_N_=0.86  *t*(10579)=-2.99  *p*=.00278 | *M*_Y_=2.87, *SD*_Y_=0.78  *M*_N_=2.93, *SD*_N_=0.75  *t*(10531)=1.89  *p*=.05863 | *M*_Y_=2.63, *SD*_Y_=0.64  *M*_N_=2.60, *SD*_N_=0.63  *t*(10467)=-1.05  *p*=.29418 | *M*_Y_=2.65, *SD*_Y_=0.60  *M*_N_=2.67, *SD*_N_=0.60  *t*(10294)=0.61  *p*=.54344 | *M*_Y_=18.59, *SD*_Y_=5.25  *M*_N_=19.19, *SD*_N_=5.30  *t*(10183)=2.58  *p*=.01002 |
| Visited or works in nursing home/hospital (Y/N) | *M*_Y_=2.40, *SD*_Y_=0.91  *M*_N_=2.32, *SD*_N_=0.92  *t*(10824)=-2.91  *p*=.00360 | *M*_Y_=1.82, *SD*_Y_=1.00  *M*_N_=1.73, *SD*_N_=0.98  *t*(10815)=-3.13  *p*=.00173 | *M*_Y_=1.85, *SD*_Y_=1.17  *M*_N_=1.76, *SD*_N_=1.14  *t*(10784)=-2.52  *p*=.01166 | *M*_Y_=3.07, *SD*_Y_=0.84  *M*_N_=3.04, *SD*_N_=0.82  *t*(10765)=-1.32  *p*=.18723 | *M*_Y_=2.34, *SD*_Y_=0.86  *M*_N_=2.21, *SD*_N_=0.87  *t*(10636)=-5.04  *p*<.00001 | *M*_Y_=1.23, *SD*_Y_=0.88  *M*_N_=1.14, *SD*_N_=0.85  *t*(10579)=-3.55  *p*=.00039 | *M*_Y_=2.87, *SD*_Y_=0.77  *M*_N_=2.94, *SD*_N_=0.75  *t*(10532)=3.16  *p*=.00158 | *M*_Y_=2.65, *SD*_Y_=0.63  *M*_N_=2.59, *SD*_N_=0.63  *t*(10468)=-2.92  *p*=.00351 | *M*_Y_=2.66, *SD*_Y_=0.61  *M*_N_=2.66, *SD*_N_=0.60  *t*(10297)=0.53  *p*=.59740 | *M*_Y_=17.89, *SD*_Y_=5.56  *M*_N_=19.33, *SD*_N_=5.24  *t*(10185)=8.95  *p*<.00001 |
| Lost job/income due to COVID-19 (Y/N) | *M*_Y_=2.43, *SD*_Y_=0.99  *M*_N_=2.33, *SD*_N_=0.92  *t*(8609)=-2.78  *p*=.00552 | *M*_Y_=2.14, *SD*_Y_=1.03  *M*_N_=1.72, *SD*_N_=0.98  *t*(8601)=-10.60  *p*<.00001 | *M*_Y_=2.00, *SD*_Y_=1.21  *M*_N_=1.75, *SD*_N_=1.13  *t*(8575)=-5.47  *p*<.00001 | *M*_Y_=2.96, *SD*_Y_=0.93  *M*_N_=3.06, *SD*_N_=0.81  *t*(8563)=3.05  *p*=.00229 | *M*_Y_=2.60, *SD*_Y_=0.86  *M*_N_=2.21, *SD*_N_=0.86  *t*(8458)=-11.21  *p*<.00001 | *M*_Y_=2.20, *SD*_Y_=0.90  *M*_N_=1.09, *SD*_N_=0.81  *t*(8417)=-33.34  *p*<.00001 | *M*_Y_=2.93, *SD*_Y_=0.84  *M*_N_=2.92, *SD*_N_=0.75  *t*(8375)=-0.35  *p*=.72773 | *M*_Y_=2.61, *SD*_Y_=0.65  *M*_N_=2.60, *SD*_N_=0.63  *t*(8320)= -0.26  *p*=.79768 | *M*_Y_=2.63, *SD*_Y_=0.68  *M*_N_=2.67, *SD*_N_=0.60  *t*(8176)=1.44  *p*=.15075 | *M*_Y_=17.34, *SD*_Y_=5.66  *M*_N_=19.33, *SD*_N_=5.24  *t*(8083)=9.00  *p*<.00001 |
| Spouse/partner lost job/income due to COVID-19 (Y/N) | *M*_Y_=2.21, *SD*_Y_=0.94  *M*_N_=2.43, *SD*_N_=0.91  *t*(5153)=8.10  *p*<.00001 | *M*_Y_=1.56, *SD*_Y_=1.00  *M*_N_=1.84, *SD*_N_=1.00  *t*(5147)=9.69  *p*<.00001 | *M*_Y_=1.68, *SD*_Y_=1.11  *M*_N_=1.77, *SD*_N_=1.15  *t*(5159)=2.65  *p*=.00805 | *M*_Y_=3.04, *SD*_Y_=0.82  *M*_N_=3.07, *SD*_N_=0.81  *t*(5153)=1.29  *p*=.19804 | *M*_Y_=2.07, *SD*_Y_=0.89  *M*_N_=2.29, *SD*_N_=0.86  *t*(5079)=8.69  *p*<.00001 | *M*_Y_=1.20, *SD*_Y_=0.85  *M*_N_=1.23, *SD*_N_=0.88  *t*(5044)=0.98  *p*=.32733 | *M*_Y_=2.88, *SD*_Y_=0.77  *M*_N_=2.98, *SD*_N_=0.75  *t*(5020)=4.46  *p*=.00001 | *M*_Y_=2.54, *SD*_Y_=0.64  *M*_N_=2.65, *SD*_N_=0.63  *t*(4985)=5.87  *p*<.00001 | *M*_Y_=2.62, *SD*_Y_=0.62  *M*_N_=2.72, *SD*_N_=0.61  *t*(4888)=5.44  *p*<.00001 | *M*_Y_=20.14, *SD*_Y_=5.07  *M*_N_=19.01, *SD*_N_=5.24  *t*(4832)=-7.24  *p*<.00001 |
| Decided not to attend an in-person general medical appointment (Y/N) | *M*_Y_=2.52, *SD*_Y_=0.87  *M*_N_=2.48, *SD*_N_=0.89  *t*(4896)=-0.63  *p*=.53036 | *M*_Y_=1.91, *SD*_Y_=0.97  *M*_N_=1.95, *SD*_N_=1.06  *t*(4891)=0.55  *p*=.58134 | *M*_Y_=2.02, *SD*_Y_=1.12  *M*_N_=1.62, *SD*_N_=1.15  *t*(4891)=-4.90  *p*<.00001 | *M*_Y_=3.03, *SD*_Y_=0.82  *M*_N_=2.88, *SD*_N_=0.82  *t*(4882)=-2.56  *p*=.01051 | *M*_Y_=2.37, *SD*_Y_=0.84  *M*_N_=2.28, *SD*_N_=0.86  *t*(4826)=-1.52  *p*=.12890 | *M*_Y_=1.23, *SD*_Y_=0.88  *M*_N_=1.32, *SD*_N_=0.93  *t*(4796)=1.42  *p*=.15686 | *M*_Y_=2.97, *SD*_Y_=0.74  *M*_N_=2.95, *SD*_N_=0.68  *t*(4780)=-0.38  *p*=.70577 | *M*_Y_=2.61, *SD*_Y_=0.63  *M*_N_=2.62, *SD*_N_=0.71  *t*(4757)=0.03  *p*=.97398 | *M*_Y_=2.67, *SD*_Y_=0.61  *M*_N_=2.71, *SD*_N_=0.55  *t*(4682)=0.87  *p*=.38706 | *M*_Y_=18.50, *SD*_Y_=5.43  *M*_N_=19.02, *SD*_N_=5.37  *t*(4632)=1.31  *p*=.18896 |
| Decided not to attend an in-person cancer care appointment (Y/N) | *M*_Y_=2.48, *SD*_Y_=0.89  *M*_N_=2.56, *SD*_N_=0.87  *t*(3548)=1.39  *p*=.16509 | *M*_Y_=1.86, *SD*_Y_=0.98  *M*_N_=2.00, *SD*_N_=1.03  *t*(3548)=2.29  *p*=.02221 | *M*_Y_=2.06, *SD*_Y_=1.15  *M*_N_=1.73, *SD*_N_=1.14  *t*(3555)= -4.72  *p*<.00001 | *M*_Y_=3.06, *SD*_Y_=0.84  *M*_N_=2.88, *SD*_N_=0.79  *t*(3553)=-3.48  *p*=.00050 | *M*_Y_=2.33, *SD*_Y_=0.86  *M*_N_=2.40, *SD*_N_=0.88  *t*(3508)=1.34  *p*=.18181 | *M*_Y_=1.23, *SD*_Y_=0.88  *M*_N_=1.31, *SD*_N_=0.95  *t*(3488)=1.62  *p*=.10586 | *M*_Y_=2.96, *SD*_Y_=0.73  *M*_N_=2.93, *SD*_N_=0.68  *t*(3477)=-0.69  *p*=.49045 | *M*_Y_=2.63, *SD*_Y_=0.62  *M*_N_=2.63, *SD*_N_=0.71  *t*(3462)=0.03  *p*=.97361 | *M*_Y_=2.67, *SD*_Y_=0.61  *M*_N_=2.74, *SD*_N_=0.59  *t*(3406)=1.82  *p*=.06899 | *M*_Y_=18.25, *SD*_Y_=5.53  *M*_N_=19.25, *SD*_N_=5.16  *t*(3373)=2.92  *p*=.00350 |
| Decided not to seek emergency/urgent care (Y/N) | *M*_Y_=2.60, *SD*_Y_=0.90  *M*_N_=2.56, *SD*_N_=0.86  *t*(1201)=-0.81  *p*=.41877 | *M*_Y_=2.10, *SD*_Y_=1.02  *M*_N_=2.01, *SD*_N_=1.00  *t*(1201)=-1.48  *p*=.13941 | *M*_Y_=2.14, *SD*_Y_=1.18  *M*_N_=1.73, *SD*_N_=1.12  *t*(1201)=-5.57  *p*<.00001 | *M*_Y_=2.99, *SD*_Y_=0.86  *M*_N_=2.91, *SD*_N_=0.81  *t*(1198)=-1.54  *p*=.12464 | *M*_Y_=2.52, *SD*_Y_=0.88  *M*_N_=2.42, *SD*_N_=0.85  *t*(1186)=-1.81  *p*=.07056 | *M*_Y_=1.46, *SD*_Y_=0.98  *M*_N_=1.29, *SD*_N_=0.92  *t*(1180)=-2.84  *p*=.00459 | *M*_Y_=3.05, *SD*_Y_=0.74  *M*_N_=2.91, *SD*_N_=0.67  *t*(1176)=-3.12  *p*=.00186 | *M*_Y_=2.65, *SD*_Y_=0.66  *M*_N_=2.62, *SD*_N_=0.69  *t*(1169)=-0.83  *p*=.40635 | *M*_Y_=2.62, *SD*_Y_=0.68  *M*_N_=2.75, *SD*_N_=0.56  *t*(1156)=3.27  *p*=.00112 | *M*_Y_=16.51, *SD*_Y_=6.13  *M*_N_=19.10, *SD*_N_=5.15  *t*(1136)=6.88  *p*<.00001 |
|  |  |  |  |  |  |  |  |  |  |  |
| Working from home (H) vs. commuting to work (C) | *M*_H_=2.28, *SD*_H_=0.96  *M*_C_=2.41, *SD*_C_=0.89  *t*(5016)=-4.91  *p*<.00001 | *M*_H_=1.65, *SD*_H_=1.03  *M*_C_=1.80, *SD*_C_=0.97  *t*(5009)=-5.48  *p*<.00001 | *M*_H_=1.74, *SD*_H_=1.13  *M*_C_=1.74, *SD*_C_=1.14  *t*(5002)=-0.03  *p*=.97789 | *M*_H_=3.05, *SD*_H_=0.82  *M*_C_=3.09, *SD*_C_=0.81  *t*(4999)=-1.91  *p*=.05568 | *M*_H_=2.13, *SD*_H_=0.93  *M*_C_=2.27, *SD*_C_=0.83  *t*(4926)=-5.56  *p*<.00001 | *M*_H_=1.30, *SD*_H_=0.90  *M*_C_=1.16, *SD*_C_=0.83  *t*(4887)=5.86  *p*<.00001 | *M*_H_=2.94, *SD*_H_=0.77  *M*_C_=2.95, *SD*_C_=0.75  *t*(4859)=-0.06  *p*=.95020 | *M*_H_=2.57, *SD*_H_=0.66  *M*_C_=2.64, *SD*_C_=0.61  *t*(4828)=-4.26  *p*=.00002 | *M*_H_=2.64, *SD*_H_=0.64  *M*_C_=2.72, *SD*_C_=0.59  *t*(4735)=-4.50  *p*=.00001 | *M*_H_=19.43, *SD*_H_=5.35  *M*_C_=19.47, *SD*_C_=4.96  *t*(4680)=-0.29  *p*=.77102 |
|  |  |  |  |  |  |  |  |  |  |  |
| Household income decreased (D) vs. other (O; i.e., same or increased) | *M*_D_=2.46, *SD*_D_=0.95  *M*_O_=2.29, *SD*_O_=0.91  *t*(10787)=-8.15  *p*<.00001 | *M*_D_=2.02, *SD*_D_=0.99  *M*_O_=1.65, *SD*_O_=0.96  *t*(10779)=-16.94  *p*<.00001 | *M*_D_=1.98, *SD*_D_=1.17  *M*_O_=1.71, *SD*_O_=1.12  *t*(10748)=-10.47  *p*<.00001 | *M*_D_=3.01, *SD*_D_=0.86  *M*_O_=3.06, *SD*_O_=0.81  *t*(10730)=2.77  *p*=.00559 | *M*_D_=2.49, *SD*_D_=0.84  *M*_O_=2.15, *SD*_O_=0.86  *t*(10599)=-17.80  *p*<.00001 | *M*_D_=1.81, *SD*_D_=0.88  *M*_O_=0.94, *SD*_O_=0.74  *t*(10545)=-49.32  *p*<.00001 | *M*_D_=2.94, *SD*_D_=0.79  *M*_O_=2.92, *SD*_O_=0.74  *t*(10496)=-1.12  *p*=.26480 | *M*_D_=2.60, *SD*_D_=0.63  *M*_O_=2.60, *SD*_O_=0.63  *t*(10431)=-0.02  *p*=.98539 | *M*_D_=2.65, *SD*_D_=0.64  *M*_O_=2.67, *SD*_O_=0.59  *t*(10259)=1.40  *p*=.16042 | *M*_D_=17.72, *SD*_D_=5.64  *M*_O_=19.59, *SD*_O_=5.12  *t*(10147)=15.35  *p*<.00001 |
|  |  |  |  |  |  |  |  |  |  |  |
| Number of telehealth appointments for general medical care | *B*=0.04, *SE*=0.01  *p*<.00001 | *B*=0.08, *SE*=0.01  *p*<.00001 | *B*=0.05, *SE*=0.01  *p*<.00001 | *B*=-0.01, *SE*=0.01  *p*=.01513 | *B*=0.06, *SE*=0.01  *p*<.00001 | *B*=0.04, *SE*=0.01  *p*<.00001 | *B*=-0.01, *SE*=0.01  *p*=.07908 | *B*=0.01, *SE*=0.004  *p*=.23808 | *B*=-0.01, *SE*=0.004  *p*=.00403 | *B*=-0.41, *SE*=0.04  *p*<.00001 |
| Number of telehealth appointments for cancer care | *B*=0.02, *SE*=0.01  *p*=.00730 | *B*=0.01, *SE*=0.01  *p*=.26728 | *B*=-0.02, *SE*=0.01  *p*=.02985 | *B*=0.02, *SE*=0.01  *p*=.00001 | *B*=0.01, *SE*=0.01  *p*=.09152 | *B*=0.01, *SE*=0.01  *p*=.14699 | *B*=0.01, *SE*=0.01  *p*=.15674 | *B*=0.02, *SE*=0.004  *p*<.00001 | *B*=0.003, *SE*=0.004  *p*=.48992 | *B*=-0.32, *SE*=0.04  *p*<.00001 |
| Total number of telehealth appointments | *B*=0.03, *SE*=0.01  *p*<.00001 | *B*=0.04, *SE*=0.01  *p*<.00001 | *B*=0.02, *SE*=0.01  *p*=.00204 | *B*=0.01, *SE*=0.01  *p*=.09499 | *B*=0.04, *SE*=0.004  *p*<.00001 | *B*=0.03, *SE*=0.01  *p*<.00001 | *B*=-0.003, *SE*=0.004  *p*=.49112 | *B*=0.01, *SE*=0.003  *p*=.00002 | *B*=-0.01, *SE*=0.003  *p*=.08740 | *B*=-0.40, *SE*=0.03  *p*<.00001 |
| Satisfaction with general medical care telehealth | *B*=-0.01, *SE*=0.01  *p*=.31943 | *B*=-0.07, *SE*=0.01  *p*<.00001 | *B*=-0.15, *SE*=0.01  *p*<.00001 | *B*=0.14, *SE*=0.01  *p*<.00001 | *B*=-0.06, *SE*=0.01  *p*<.00001 | *B*=-0.09, *SE*=0.01  *p*<.00001 | *B*=0.07, *SE*=0.01  *p*<.00001 | *B*=0.06, *SE*=0.01  *p*<.00001 | *B*=0.07, *SE*=0.01  *p*<.00001 | *B*=0.56, *SE*=0.07  *p*<.00001 |
| Satisfaction with cancer care telehealth | *B*=0.02, *SE*=0.01  *p*=.15407 | *B*=-0.10, *SE*=0.01  *p*<.00001 | *B*=-0.16, *SE*=0.01  *p*<.00001 | *B*=0.15, *SE*=0.01  *p*<.00001 | *B*=-0.05, *SE*=0.01  *p*<.00001 | *B*=-0.09, *SE*=0.01  *p*<.00001 | *B*=0.09, *SE*=0.01  *p*<.00001 | *B*=0.07, *SE*=0.01  *p*<.00001 | *B*=0.07, *SE*=0.01  *p*<.00001 | *B*=0.62, *SE*=0.07  *p*<.00001 |

*Note.* Cells highlighted in green indicate significant effects (*p*<.0002 after Bonferroni correction); non-highlighted cells indicate non-significant effects (*p*≥.0002).
